# Structural and Functional Analysis of DDX41: a bispecific immune receptor for DNA and cyclic dinucleotide

**DOI:** 10.1038/srep34756

**Published:** 2016-10-10

**Authors:** Hiroki Omura, Daisuke Oikawa, Takanori Nakane, Megumi Kato, Ryohei Ishii, Ryuichiro Ishitani, Fuminori Tokunaga, Osamu Nureki

**Affiliations:** 1 Department of Biophysics and Biochemistry, Graduate School of Science, The University of Tokyo, 2-11-16 Yayoi, Bunkyo-ku, Tokyo 113-0032, Japan; 2 Laboratory of Molecular Cell Biology, Institute for Molecular and Cellular Regulation, Gunma University, 3-39-15 Showa-machi, Maebashi, Gunma 371-8512, Japan; 3 Department of Pathobiochemistry, Osaka City University, Graduate School of Medicine, 1-4-3 Asahi-machi, Abeno-ku, Osaka 545-8585, Japan

## Abstract

In the innate immune system, pattern recognition receptors (PRRs) specifically recognize ligands derived from bacteria or viruses, to trigger the responsible downstream pathways. DEAD box protein 41 (DDX41) is an intracellular PRR that triggers the downstream pathway involving the adapter STING, the kinase TBK1, and the transcription factor IRF3, to activate the type I interferon response. DDX41 is unique in that it recognizes two different ligands; *i.e*., double-stranded DNA (dsDNA) and cyclic dinucleotides (CDN), *via* its DEAD domain. However, the structural basis for the ligand recognition by the DDX41 DEAD domain has remained elusive. Here, we report two crystal structures of the DDX41 DEAD domain in apo forms, at 1.5 and 2.2 Å resolutions. A comparison of the two crystal structures revealed the flexibility in the ATP binding site, suggesting its formation upon ATP binding. Structure-guided functional analyses *in vitro* and *in vivo* demonstrated the overlapped binding surface for dsDNA and CDN, which is distinct from the ATP-binding site. We propose that the structural rearrangement of the ATP binding site is crucial for the release of ADP, enabling the fast turnover of DDX41 for the dsDNA/CDN-induced STING activation pathway.

The innate immune system functions as the first-line of defense against invading bacteria and viruses. In this system, proteins called pattern recognition receptors (PRRs) recognize pathogen-associated molecular patterns (PAMPs) as ligands, and finally induce the production of interferons or inflammatory cytokines^1^. Several cytosolic PRRs that recognize RNA, DNA or cyclic dinucleotides (CDN), including cyclic di-GMP and cyclic di-AMP^2^,^3^, have been reported. Almost all of the PRRs share the common feature of specifically recognizing a single type of PAMP. In contrast, DEAD box protein 41 (DDX41), a member of the helicase superfamily 2 (SF2), is unique in that it can recognize two different types of ligands, double-stranded DNA (dsDNA) and CDN^4^,^5^.

DDX41 consists of two RecA-like domains (*i.e*., DEAD and HELICc domains), which are conserved among DEAD box proteins. These domains have several conserved motifs, such as the Q motif and motif I, which are both essential for ATP binding by the DEAD box proteins^6^. A previous study showed that DDX41 containing the DEAD and HELICc domains has the ATPase activity^7^. The crystal structure of the DDX41 HELICc domain revealed its structural similarity to the HELICc domains of other DEAD box proteins^8^.

In the signaling pathway of the DDX41-related innate immune responses, previous reports demonstrated that its HELICc domain is phosphorylated by Bruton’s tyrosine kinase (BTK)^9^, and then it recognizes dsDNA and/or CDN via its DEAD domain^4^,^5^. After the ligand recognition, DDX41 binds to STING (stimulator of interferon genes) located on the endoplasmic reticulum membrane^4^,^5^. STING subsequently promotes the phosphorylation of TBK1 (TANK-Binding Kinase 1) and IRF3 (interferon regulatory transcription factor 3), which finally induce the production of type I interferon^10^,^11^.

To understand the molecular mechanism of this signaling pathway, the structural basis for the mechanism of dsDNA and CDN recognition by the DDX41 DEAD domain is required. Although previous studies showed that dsDNA disturbs the DDX41–CDN interaction^5^, the binding site of the ligands and the mechanism by which DDX41 recognizes them have remained elusive. In this work, we determined two different crystal structures of the DDX41 DEAD domain in apo forms. Comparisons between these two structures and the AMPPNP-bound structure of another DEAD box protein revealed the flexibility in the ATP binding site, suggesting its formation upon ATP binding. In addition, structure-guided functional analyses revealed the residues critical for dsDNA and CDN recognition, and suggested the putative dsDNA/CDN-binding surface.

## Results

### The DDX41 DEAD domain recognizes both dsDNA and CDN

**Figure 1:**
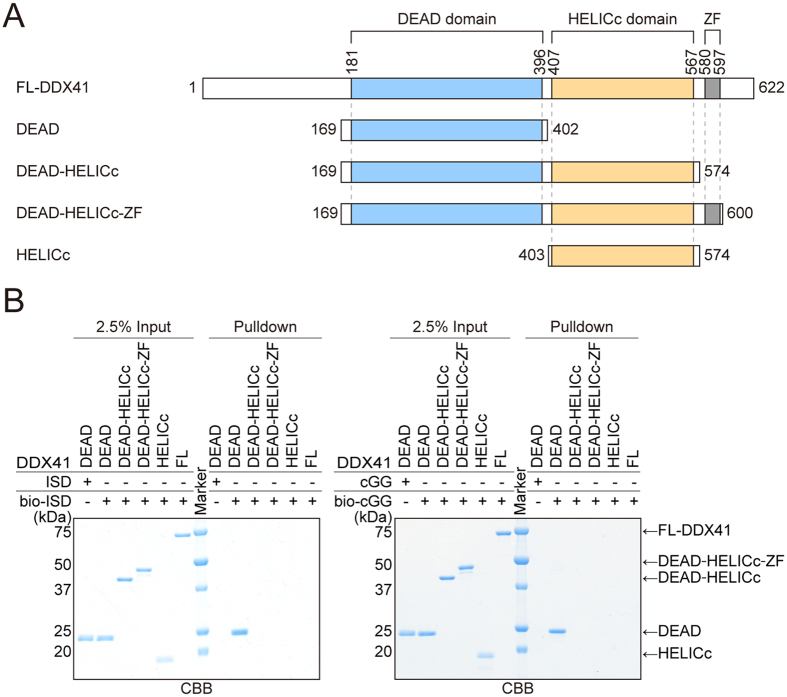
The DDX41 DEAD domain binds to dsDNA and CDN. (**A**) FL-DDX41 and its truncated variants. DDX41 contains the DEAD domain (sky blue), the HELICc domain (orange) and the Zinc finger (ZF, gray). (**B**) Pull-down assays of DDX41, using bio-ISD (left panel) and bio-cGG (right panel). In all of the pull-down assays, the final concentration of bio-ISD, bio-cGG, bio-ATP or bio-AMPPNP is 1 μM, unless otherwise stated. Gels were run under the same experimental conditions and are shown as cropped gels. Full-length gels with indicated cropping lines are shown in Figure S7.

To understand the ligand recognition mechanism of DDX41, we tested the dsDNA- and CDN-binding abilities of DDX41 and its variants. Previous studies reported that full-length DDX41 (FL-DDX41) and its truncated variants containing the DEAD domain (DEAD and DEAD-HELICc; Fig. 1A) can bind to biotinylated dsDNA and CDN^4^,^5^. Therefore, we examined the dsDNA- and CDN-binding abilities of FL-DDX41 and its truncated variants (DEAD, DEAD-HELICc, DEAD-HELICc-ZF, and HELICc; Fig. 1A) by pull-down assays, using 5′-biotinylated immune stimulatory DNA (bio-ISD) or biotinylated cyclic di-GMP (bio-cGG). The results showed that DEAD can bind to both ISD and cGG, whereas neither DEAD-HELICc, DEAD-HELICc-ZF nor FL-DDX41 bound to either ISD or cGG (Fig. 1B). These results indicated that the DEAD domain itself can bind to both dsDNA and CDN, and its binding activity is inhibited by the presence of the HELICc domain *in vitro*.

To further assess the possibility that the HELICc domain inhibits the dsDNA- and CDN-binding activities of the DEAD domain, we tested the effect of the isolated HELICc domain on the dsDNA- and CDN-binding activities of DEAD. The results showed that increasing amounts of HELICc did not affect the dsDNA- and CDN-binding activities of DEAD (Figure S1A). These results suggested that the linker between the DEAD and HELICc domains is important for the inhibitory activity. This linkage anchors the HELICc domain to raise its local concentration around the DEAD domain, where it may mask the dsDNA- and CDN-binding surface on the DEAD domain.

Previous studies reported that the BTK-dependent phosphorylation of the HELICc domain is crucial for the activation and ligand recognition of DDX41^9^. To clarify the inhibition mechanism of the dsDNA- and CDN-binding activities by the HELICc domain, we examined the ligand-binding abilities of phosphorylated FL-DDX41 by the pull-down assay. However, the results showed that phosphorylated FL-DDX41 bound neither dsDNA nor CDN, regardless of the presence and absence of ATP, ADP and AMPPNP (Figure S1B and S1C).

### DDX41 recognizes dsDNA and CDN at the same binding site

**Figure 2:**
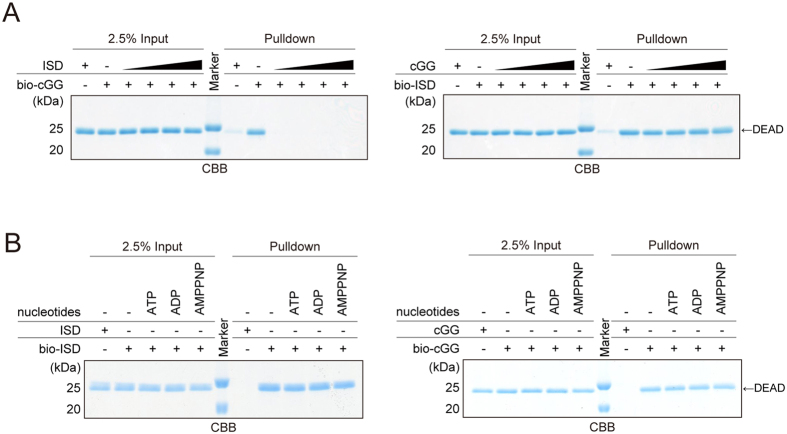
dsDNA and CDN bind to the DEAD domain at the same sites. (**A**) Pull-down assays of DEAD, using bio-cGG in the presence of unlabeled ISD (left panel), and bio-ISD in the presence of unlabeled cGG (right panel). The final concentrations of unlabeled ISD or cGG are 0, 0.5, 1, 2 or 4 μM. (**B**) Pull-down assays of DEAD, using bio-ISD (left panel) or bio-cGG (right panel), in the presence of 1 mM unlabeled ATP, ADP or AMPPNP. Gels were run under the same experimental conditions and are shown as cropped gels. Full-length gels with indicated cropping lines are shown in Figure S7.

To further explore the dsDNA, CDN and ATP recognition mechanism by DDX41, we examined whether dsDNA binding to the DEAD domain is affected by CDN, and *vice versa*, using the pull-down assay. The results revealed that the interaction between DEAD and bio-cGG was strongly impaired by the presence of increasing amounts of unlabeled ISD (Fig. 2A, left panel), indicating that dsDNA binding competes with CDN binding to the DDX41 DEAD domain. In contrast, the interaction between DEAD and bio-ISD was not affected by the presence of increasing amounts of unlabeled cGG (Fig. 2A, right panel). Thus, these results suggested that both dsDNA and CDN are recognized at the same ligand-binding site of the DEAD domain, which has higher affinity for dsDNA than CDN.

The DEAD box proteins typically bind to ATP via the conserved ATP-binding site, which is mainly located in the DEAD domain^6^. Several crystal structures of other DEAD box proteins bound to ADP, AMP or AMPPNP have been reported. Thus, we first examined the ATP-binding ability of the DEAD domain by the pull-down assay, using biotinylated ATP (bio-ATP) or biotinylated AMPPNP (bio-AMPPNP), and determined that DEAD binds to both bio-ATP and bio-AMPPNP (Figure S1D). Next, we examined whether ATP, ADP, and AMPPNP affect the binding of dsDNA and CDN to the DEAD domain, and *vice versa*. We found that the presence of ATP, ADP or AMPPNP had no effect on both the bio-cGG and bio-ISD binding (Fig. 2B). Similarly, the presence of unlabeled cGG had no effect on both the bio-ATP and bio-AMPPNP binding (Figure S1E). Collectively, these results suggested that the recognition sites for dsDNA and CDN are different from that for ATP, even though ATP and CDN share common structural features, including the base, ribose, and phosphate moieties.

### Overall structures of the DDX41 DEAD domain

**Figure 3:**
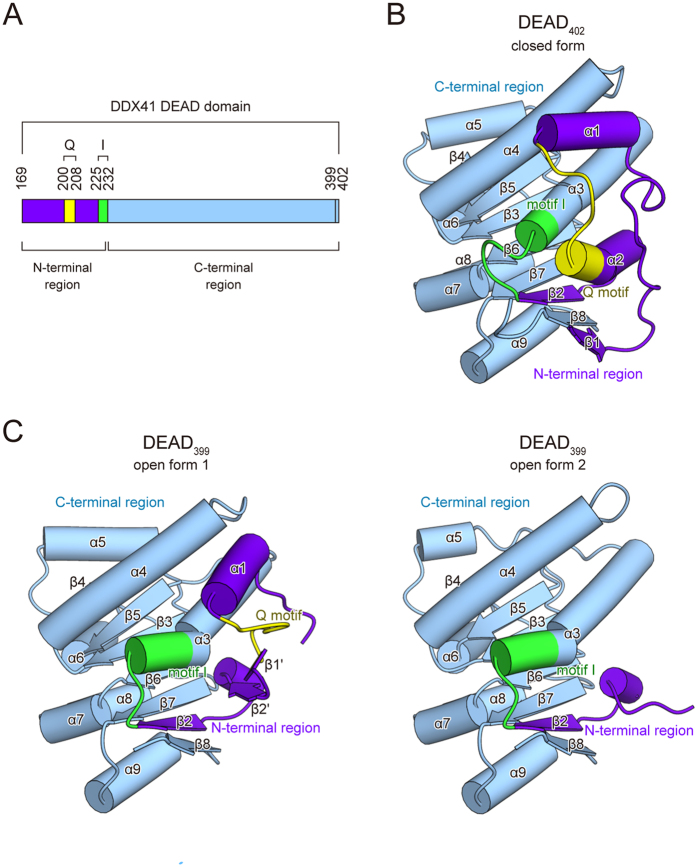
Overall structures of the DEAD domain. (**A**) The motif organization of the DEAD domain. The DEAD domain contains the N-terminal region (violet), the C-terminal region (sky blue), the Q motif (yellow) and motif I (green). The color codes representing the motifs and the N- or C-terminal regions are used similarly in the other figures, unless otherwise stated. (**B**) The overall structure of the closed form. (**C**) The overall structures of the open form 1 (left panel) and the open form 2 (right panel).

To obtain insights into the dsDNA- and CDN-recognition mechanism of DDX41, we sought to solve the crystal structures of the human DDX41 DEAD domain. We determined the apo-form structures of two constructs, residues 169–402 (DEAD_402_) and 169–399 (DEAD_399_), at 1.5 and 2.2 Å resolutions, respectively (Fig. 3A–C). Both structures adopt a RecA-like fold with one core β-sheet surrounded by α-helices, and their C-terminal regions (233–399) have almost the same conformation, with an RMSD of 1.16 Å over 161 Cα atoms (Fig. 3B,C). Thus, when we focus on the C-terminal region, we discuss the DEAD_402_ structure, since it was determined at higher resolution. In contrast, the conformations of the N-terminal region (169–232) are different in the crystal structures. In the crystal structure of DEAD_402_, helix α2 and the Q motif are located adjacent to helix α3 and motif I, and the N-terminal residues (169–173) form a β strand (β1) and participate in the core β-sheet formation (Fig. 3B). We hereafter refer to this conformation of the N-terminal region as the “closed” form. In contrast to DEAD_402_, the crystal structure of DEAD_399_ contains four molecules in the asymmetric unit, and their N-terminal regions adopt two different conformations (Fig. 3C). In one conformation, the Q motif is located away from α3 and motif I. Helix α2 is rearranged to form two β strands (β1′ and β2′), and residues 169–183 are disordered (Fig. 3C, left panel). We hereafter refer to this conformation of the N-terminal region as the “open form 1”. In the other conformation, helix α3 and motif I adopt similar conformations to those in open form 1, while residues 169–209, including the entire Q motif, are disordered (Fig. 3C, right panel). We hereafter refer to this conformation of the N-terminal region as the “open form 2”.

### Structural rearrangement of the ATP-binding site of the DDX41 DEAD domain

Several crystal structures of DEAD box proteins in the ADP-, AMP- or AMPPNP-bound forms have been reported, and suggested that the important motifs for ATP recognition, including the Q motif and motif I, are structurally conserved^8^. Among the DEAD box proteins, the amino-acid sequence of DDX3 is most similar to that of DDX41. To understand the ATP recognition mechanism by the DDX41 DEAD domain, we compared the present crystal structures with that of the DDX3 DEAD domain in the AMPPNP-bound form (PDB ID: 5E7M).

**Figure 4:**
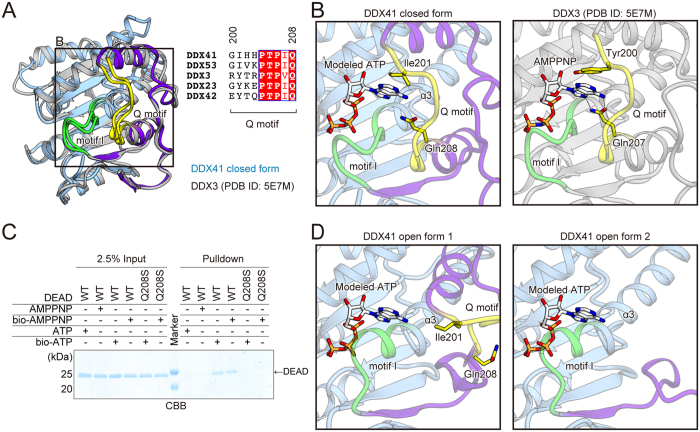
Three forms of DDX41 reveal the structural rearrangement in the ATP-binding site. (**A**) Structure and sequence comparison between DDX41 and DDX3 (PDB ID: 5E7M). The Q motif and motif I of DDX3 are colored as in DDX41, whereas the other regions are colored silver. The amino-acid sequences of full-length DDX23 and DDX42 are the second and third most similar to that of DDX41. (**B**) Docking model of the DDX41 closed form bound to ATP (left panel), based on the DDX3-AMPPNP complex structure (PDB ID: 5E7M) (right panel). (**C**) Pull-down assays of the DEAD mutant, using bio-ATP and bio-AMPPNP. A cropped gel is shown. The full-length gel with the cropping lines indicated is shown in Figure S7. (**D**) Docking models of the DDX41 open forms bound to ATP, based on the DDX3-AMPPNP complex structure (PDB ID: 5E7M). Models of the open form 1 (left panel) and the open form 2 (right panel) are shown.

The closed form of the DDX41 DEAD domain superimposes well on that of DDX3, with an RMSD of 1.56 Å over 215 Cα atoms. Especially, this superimposition revealed that the structure around the ATP-binding site, including the Q motif and motif I, adopts a similar conformation to that of DDX3 (Fig. 4A). This similarity between these proteins allowed us to create a docking model of the DDX41-ATP complex, based on the DDX3-AMPPNP complex structure (Fig. 4B). In the crystal structure of the DDX3-AMPPNP complex, Tyr200 recognizes the adenine base of AMPPNP via a stacking interaction, while the corresponding residue is Ile (Ile201) in DDX41, suggesting that the adenine base is recognized by a hydrophobic interaction in DDX41 (Fig. 4B). Similar base recognition by an Ile residue was also observed in the crystal structure of the DDX53-AMP complex, supporting our structural model (Fig. 4A, PDB ID: 3IUY^8^). Furthermore, our structural model suggested that Gln208 in the Q motif of DDX41 recognizes the adenine moiety in a base-specific manner, similar to Gln207 in DDX3 (Fig. 4B, right panel). The Q208S mutant of DEAD exhibited decreased ATP-binding ability as compared to the wild-type protein (Fig. 4C), suggesting the importance of Gln208 for the nucleotide binding. Furthermore, the model suggested that the main chain amide groups and Lys231 in motif I extensively recognize the β- and γ-phosphate groups of ATP, in a similar manner to those of motif I in DDX3 (Fig. 4B, S2A and S2B). Interestingly, a malic acid molecule from the crystallization solution is present at this putative binding site for the ATP phosphate group in the closed form structure (Figure S3). One of the two carboxylate groups of the malic acid interacts with the main chain amide groups of motif I, while the other carboxylate group forms a salt bridge with the Arg residues in the adjacent molecule in the crystalline lattice. This observation is consistent with the fact that the crystals of the closed form were obtained only under conditions containing malic acid.

Next, we created docking models of ATP-bound DDX41, based on the open form structures (Fig. 4D). The models suggested that the structural rearrangement and disorder observed in the open forms 1 and 2 may affect the ATP binding by the DEAD domain. In these models, the Q motif, including Ile201 and Gln208, is distant from the modeled ATP in the open form 1, while it is disordered in the open form 2 (Fig. 4D). Moreover, in both open forms 1 and 2, motif I forms part of the extended α3 helix, which sterically clashes with the modeled ATP (Fig. 4D, S2C and S2D). Consequently, the structure of the ATP-binding site is completely rearranged in these open forms, and thus they do not seem to be able to bind ATP. Therefore, these results suggested that the closed form represents the ATP-bound state of DDX41, while both open forms 1 and 2 represent its nucleotide-free state.

### Putative dsDNA and CDN binding sites of DDX41

**Figure 5:**
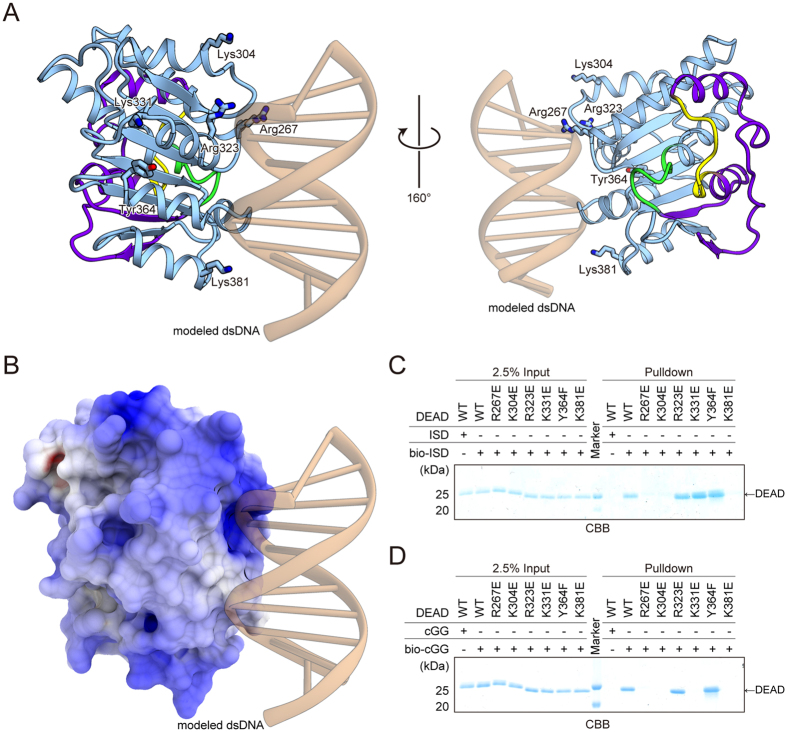
Putative dsDNA and CDN binding sites of DDX41. (**A**) Docking model of the DDX41 DEAD domain bound to dsDNA. The dsDNA is colored brown. (**B**) Electrostatic surface potentials of the DDX41 closed form (contoured from −10 kT e^−1^ [red] to +10 kT e^−1^ [blue]). (**C**) Pull-down assays of the DEAD mutants and bio-ISD. (**D**) Pull-down assays of the DEAD mutants and bio-cGG. Gels were run under the same experimental conditions and are shown as cropped gels. Full-length gels with the cropping lines indicated are shown in Figure S7.

To obtain structural insight into its dsDNA-binding mechanism, we created a model structure of the dsDNA-bound DEAD domain of DDX41. Among the SF2 proteins with structures reported in complex with dsDNA, the RecA-like domain of Rad54^12^ shares the highest structural similarity (23.9% sequence identity) with the DDX41 DEAD domain. The closed form of the DDX41 DEAD domain was superimposed on that of Rad54, with an RMSD of 2.36 Å over 157 Cα atoms. In addition, a previous study^4^, as well as our data (Fig. 1B), indicated that the HELICc domain of DDX41 is not directly involved in the dsDNA binding. Thus, we created the docking model of the DDX41 DEAD domain bound to dsDNA, based on the Rad54 structure (PDB ID: 1Z63) (Fig. 5A,B). The model suggested that the dsDNA binding site is located around the surface of the C-terminal region, and involves Arg267, Lys304, Tyr364 and Lys381 (Fig. 5A). To corroborate this putative dsDNA-binding site, we examined the effects of mutations in this site on the dsDNA-binding ability, by the pull-down assay using bio-ISD. The results showed that the R267E, K304E and K381E mutations decrease the bio-ISD binding ability of DEAD (Fig. 5C), suggesting the importance of Arg267, Lys304 and Lys381 for the dsDNA binding. Next, we examined the CDN-binding ability of these mutants, using bio-cGG. The results demonstrated that the R267E, K304E and K381E mutations also decreased the CDN-binding ability of DEAD (Fig. 5D), thus suggesting that these residues in the putative dsDNA-binding surface are involved in both dsDNA and CDN binding. In contrast, the K331E mutation exhibited decreased binding ability only for CDN and not dsDNA (Fig. 5C,D), implying that this residue is exclusively involved in CDN binding. Thus, these results suggested that the dsDNA- and CDN-binding sites of DDX41 overlap with each other. This notion is further supported by a previous report that bio-cGG binding to FL-DDX41 is affected by unlabeled dsDNA^5^. We hereafter refer to this surface around the C-terminal region as the putative dsDNA/CDN-binding surface. Furthermore, Lys304, Lys331 and Lys381 on this dsDNA/CDN-binding surface are not conserved in other paralogues of DDX41, such as DDX3, DDX23, DDX42, and DDX53 (Figure S4A), while they are highly conserved in the orthologues of DDX41 from other species (Figure S4B). These observations are consistent with the fact that the dsDNA- and CDN-binding activities are specific features of DDX41.

### The effect of DDX41 mutations on interferon production

**Figure 6:**
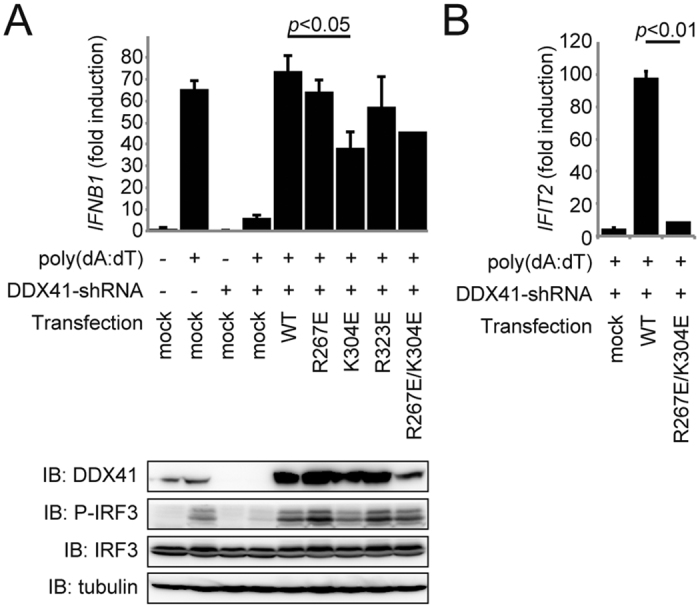
The dsDNA/CDN-binding surface of DDX41 is critical for the innate-immune responses. (**A,B**) Quantitative PCR analyses of *IFNB1* (**A**) and *IFIT2* (**B**) transcription in THP-1 cells. The cells were treated with shRNA targeting the mRNA encoding DDX41, and then transfected with vectors expressing FL-DDX41 WT or mutants. Gels were run under the same experimental conditions and are shown as cropped blots. Full-length blots with the cropping lines indicated are shown in Figure S7.

To confirm the importance of this putative dsDNA/CDN-binding surface of DDX41 for signal transduction in the innate immune system, we overexpressed the wild type or mutants of DDX41 in shRNA-mediated *DDX41*-knockdown THP1 cells, and then examined the transcription of *IFNB1* by quantitative PCR in the presence of poly(dA:dT), as described previously^5^. The wild-type DDX41 rescued both the *IFNB1*-transcription and IRF3-phosphorylation activities of the *DDX41*-knockdown THP1 cells, whereas the K304E mutant could not rescue these activities (Fig. 6A). Furthermore, we examined the effects of the DDX41 mutations on the transcription of *IFIT2* (interferon-induced protein with tetratricopeptide repeats 2) by quantitative PCR. The transcription of *IFIT2* is activated by type-I interferons^13^. As compared to wild-type DDX41, the R267E/K304E mutant could not rescue the *IFIT2*-transcription activity (Fig. 6B). Thus, Lys304 of DDX41 is crucial for the dsDNA-induced type I interferon production and the activation of the downstream pathways of type I interferons. Overall, these results suggested that the dsDNA/CDN-binding surface of DDX41 is critical for its exogenous DNA recognition, and thus important for the innate-immune responses in the cellular context.

## Discussion

DDX41 is a unique PRR that recognizes two different types of PAMPs: dsDNA and CDN^4^,^5^. In this study, we determined the crystal structures of the DDX41 DEAD domain in the closed and open forms. Previous studies reported that the mutations of residues in the DEAD domain of human DDX41, F183I, A225D, E247K, P321L and I396T, cause acute myeloid leukemia (AML) syndrome^14^. All of these residues form the inside core of the DEAD domain (Figure S5), suggesting that the mutations of these residues lead to the misfolding of DDX41, which causes AML. Based on the apo form structure, we created the model structure of dsDNA-bound DDX41 and performed the mutational analyses. These analyses revealed the residues involved in the dsDNA and CDN binding, and suggested that the DEAD domain recognizes these different ligands at overlapping sites.

A structural comparison between the open and closed forms of the DDX41 DEAD domain revealed the structural rearrangement in the N-terminal region, which drastically changes the conformation of the ATP-binding site. One of the intriguing points of these structures is that the ATP-binding site is formed in the closed form without binding an adenine nucleotide. Instead, the carboxylate group of malic acid contained in the crystallization solution is bound to the phosphate-binding site formed by motif I (Figure S3). Thus, in the present crystal structure, the DDX41 DEAD domain is trapped in the closed form by the carboxylate group of malic acid, which is bound by mimicking the phosphate group. This observation led to a putative mechanism of the structural change that occurs in the DDX41 DEAD domain upon ATP binding. The phosphate moiety of ATP binds to motif I, which induces the helix-to-loop transition of motif I (Fig. 4B,D and S2). This structural transition of motif I enables the interaction between the Q motif and motif I, including the hydrogen bonds between Thr205, Gln208 in the Q motif and Ser229, Gly230 in motif I (Figure S3). These interactions fix the Q motif, including Gln208 and Ile201, in the closed-form conformation and thus create the adenine moiety binding pocket (Fig. 4B). In the cellular context, this structural change may occur in the transition from the closed form in the ATP-bound state to the open form after the ATP hydrolysis and subsequent ADP release. What is the role of this structural change of DDX41 in the dsDNA and CDN sensing mechanism? RIG-I (DDX58), another SF2 protein that functions as a cytosolic PRR, recognizes single- and double-stranded RNA to trigger the downstream pathway for the innate immune responses^1^. Previous studies reported that ATP binding and hydrolysis and ADP release accelerate the dissociation of the bound RNA to facilitate the fast ligand recognition turnover of RIG-I^15^,^16^. Thus, it is possible that the structural transition between the open and closed forms of DDX41 also accelerates the binding and release of ligands to facilitate the fast turnover, as in the case of RIG-I.

The results of our pull-down assays using the purified proteins prepared by the *E. coli* expression system demonstrated that the DDX41 variants containing only the DEAD domain bind to dsDNA and CDN, while those containing both the DEAD and HELICc domains do not (Fig. 1B). These results suggested that the HELICc domain inhibits the interaction between the DEAD domain and these ligands. Given the crystal structure of the DDX41 HELICc domain^8^ and the length and location of the linker region between the DEAD and HELICc domains, the HELICc domain can adopt a conformation where it is located near the dsDNA/CDN-binding surface, to disturb its interactions with ligands. For example, in the DDX41 DEAD-HELICc model structure created based on the crystal structure of DDX3^17^ (Figure S6A), the dsDNA/CDN-binding surface of the DEAD domain is completely covered by the HELICc domain (Figure S6B). Furthermore, our pull-down assays demonstrated that the isolated HELICc domain does not affect the ligand binding ability of the DEAD domain, suggesting that the interaction between the DEAD and HELICc domains is weak. The linker region between the DEAD and HELICc domains restricts the location of the HELICc domain and keeps it near the dsDNA/CDN-binding surface, which may perturb the ligand binding by the DEAD domain. In contrast, previous studies, using a crude extract prepared from cultured human cells, showed that FL-DDX41 also binds to both biotinylated dsDNA and CDN^4^,^5^. Furthermore, in a luciferase reporter assay in L929 cells, the HELICc-truncated variant reportedly exhibited higher *Ifnb*-promoter induction activity upon dsDNA recognition than that of FL-DDX41^4^. Thus, these results suggest the possibility that an unidentified factor or post-translational modification disturbs the interaction between the HELICc and DEAD domains, which allows ligand access to the dsDNA/CDN-binding surface accessible for ligands. Another PRR, TLR (toll-like receptor) 4, requires the MD-2 protein as a co-factor to recognize its ligand, LPS (lipopolysaccharide)^18^,^19^. In this study, we examined the possibility that the phosphorylation by BTK is involved in the activation of FL-DDX41, but found that it had no effect on the ligand binding (Figure S1B and S1C). Overall, in conjunction with the previous reports, our present structural and functional analyses strongly suggest the existence of a regulation mechanism of DDX41, although further studies will be required to identify the regulatory factor(s).

## Materials and Methods

### Protein preparation

The gene encoding *Homo sapiens* DDX41 (residues 1–622, FL-DDX41) was inserted into the modified pET28a vector (Novagen), and then FL-DDX41 or its truncated variants (DEAD, DEAD-HELICc, DEAD-HELICc-ZF or HELICc) were subcloned into the pE-SUMOpro Kan vector (LifeSensors). Point mutations of DEAD (residues 169–402) were introduced by QuikChange Site-Directed Mutagenesis (Agilent Technologies). All DDX41 constructs were expressed as N-terminal His_6_-SUMO-tag fused proteins in *E. coli* Rosetta2 (DE3) (Novagen) cells. The cells were grown at 37 °C in LB medium to an OD_600_ of 0.8. After the induction of protein expression by 0.4 mM isopropyl-β-D-thiogalactopyranoside, the cells were further cultured at 20 °C for 18 h and then harvested by centrifugation.

All constructs of DDX41, except for HELICc, were purified in a similar manner, as follows. The harvested cells were resuspended in buffer A (50 mM HEPES-NaOH, pH 7.0, 300 mM NaCl, 20 mM imidazole, 10% glycerol, 2 mM MgCl_2_, 5 mM 2-mercaptoethanol) containing benzonase nuclease (Novagen), and were lysed by sonication. The lysates were centrifuged at 40,000 *g* for 30 min, and then the supernatants were loaded onto Ni-NTA Superflow resin (QIAGEN) packed in an Econo-Column (Bio-Rad). The DDX41-bound resin was washed with buffer A, and then the proteins were eluted with buffer B (50 mM HEPES-NaOH, pH 7.0, 300 mM NaCl, 500 mM imidazole, 10% glycerol, 2 mM MgCl_2_, 5 mM 2-mercaptoethanol). The eluted proteins were dialyzed with His-tag fused Ulp1 protease (prepared in our lab) at 4 °C for 20 h, to remove the His_6_-SUMO-tag of DDX41. The proteins were again loaded onto Ni-NTA Superflow resin (QIAGEN) packed in an Econo-Column (Bio-Rad). The flow-through and wash fractions were collected, and then were loaded onto a HiTrap Heparin HP Column (GE Healthcare), equilibrated with buffer C (50 mM HEPES-NaOH, pH 7.0, 100 mM NaCl, 10% glycerol, 2 mM MgCl_2_, 1 mM DTT). The proteins were eluted using a linear gradient of 100–500 mM NaCl. The eluted proteins were diluted, and then loaded onto Resource S column (GE Healthcare) equilibrated with buffer C. The proteins were eluted using a linear gradient of 100–500 mM NaCl, and were concentrated using Amicon Ultra Centrifugal Filter Units (MWCO 10 kDa for DEAD WT and mutants, and 30 kDa for DEAD-HELICc, DEAD-HELICc-ZF and FL-DDX41) (Millipore). The proteins were then loaded onto a Superdex 200 Increase 10/300 size exclusion column (GE Healthcare) equilibrated with buffer D (50 mM HEPES-NaOH, pH 7.5, 150 mM NaCl, 10% glycerol, 2 mM MgCl_2_, 1 mM DTT). The proteins were concentrated with Amicon Ultra Centrifugal Filter Units (Millipore), flash frozen in liquid nitrogen, and stored at −80 °C.

HELICc was purified in a similar manner to the other constructs, until the 2^nd^ Ni-NTA column step. After purification on the Ni-NTA resin, the collected HELICc was loaded onto a HiTrap Heparin HP column (GE Healthcare), equilibrated with buffer C. The unbound fraction was collected and loaded onto a Resource Q column (GE Healthcare) equilibrated with buffer C. The unbound fraction was collected and concentrated using Amicon Ultra Centrifugal Filter Units (MWCO 10 kDa) (Millipore). The protein was then loaded onto a Superdex 200 Increase 10/300 size exclusion column (GE Healthcare) equilibrated with buffer D. The protein was concentrated with Amicon Ultra Centrifugal Filter Units (MWCO 10 kDa) (Millipore), flash frozen in liquid nitrogen, and stored at −80 °C.

The gene encoding *Mus musculus* BTK (residues 214–659) was cloned into the pFastBac HTb vector (Invitrogen) to create the baculovirus. Sf9 cells were infected with the baculovirus to express the N-terminal His_6_-tag- and Tobacco Etch Virus (TEV) protease cleavage site-fused BTK. The cells were harvested by centrifugation, 48 h after infection. The harvested cells were resuspended in buffer E (50 mM Tris-HCl, pH 8.0, 300 mM NaCl, 20 mM imidazole, 5 mM 2-mercaptoethanol), containing complete protease inhibitor (Roche), and lysed by sonication. The lysate was centrifuged at 40,000 *g* for 30 min, and then the supernatant was further centrifuged at 138,000 *g* for 1 h. The supernatant was loaded onto Ni-NTA Superflow resin (QIAGEN) packed in an Econo-Column (Bio-Rad). The BTK-bound resin was washed with buffer E, and then the protein was eluted with buffer F (50 mM Tris-HCl, pH 8.0, 300 mM NaCl, 500 mM imidazole, 5 mM 2-mercaptoethanol). The eluted protein was dialyzed with His-tag fused TEV protease (prepared in our lab) at 4 °C for 20 h, to remove the His_6_-tag of BTK. The protein was loaded again onto Ni-NTA Superflow resin (QIAGEN) packed in an Econo-Column (Bio-Rad). The flow-through and wash fractions were collected and loaded onto a Resource Q column (GE Healthcare), equilibrated with buffer G (20 mM Tris-HCl, pH 8.5, 20 mM NaCl, 1 mM DTT). The protein was eluted as two separate peaks, using a linear gradient of 20–500 mM NaCl. These two peaks correspond to the monomer and dimer of BTK, as previously described^20^. The fractions from the two peaks were collected separately, and were concentrated using Amicon Ultra Centrifugal Filter Units (MWCO 10 kDa) (Millipore). The protein was loaded onto a Superdex 200 Increase 10/300 size exclusion column (GE Healthcare) equilibrated with buffer H (20 mM Tris-HCl, pH 7.5, 150 mM NaCl, 1 mM DTT). The protein was concentrated with Amicon Ultra Centrifugal Filter Units (MWCO 10 kDa) (Millipore), flash frozen in liquid nitrogen, and stored at −80 °C.

### Crystallization

Two DDX41 constructs, DEAD_402_ (residues 169–402) and DEAD_399_ (residues 169–399), were used for crystallization. Before crystallization, DEAD_402_ and DEAD_399_ were diluted to 15 mg ml^−1^ and 7.5 mg ml^−1^, respectively. DEAD_402_ was then incubated with 5 mM cyclic di-GMP for 1 h at 4 °C. After the incubation, DEAD_402_ was highly precipitated. The precipitate was removed with an Ultrafree-MC Centrifugal Hydrophilic Filter Unit (Millipore). Crystallization trials were performed by the sitting drop vapor diffusion method at 4 °C, using a Mosquito crystallization robot (TTP Labtech). Crystals of DEAD_402_ and DEAD_399_ were obtained with reservoir solution A (2.1 M DL-malic acid, pH 7.0) and reservoir solution B (0.18 M tri-ammonium citrate, 20% (w/v) polyethylene glycol 3350), respectively. The reservoir solutions supplemented with 5% and 25% glycerol were used as cryoprotectants for DEAD_402_ and DEAD_399_, respectively.

### Data collection, structure determination and refinement

Diffraction data sets of DEAD_402_ and DEAD_399_ were collected at the Swiss Lightsource PXII and SPring-8 BL32XU, respectively. The data sets of DEAD_402_ were processed with the programs XDS^21^. The data sets of DEAD_399_ were processed with MOSFLM^22^, FECKLESS, POINTLESS and AIMLESS^23^. The DEAD_399_ crystals exhibited non-merohedral twinning. Two overlapping lattices were identified by the multiple-lattice indexing algorithm in MOSFLM and integrated separately, combined by FECKLESS, and scaled with AIMLESS. The phases of DEAD_402_ were determined with the program Phaser^24^. The search model for DEAD_402_ was created, based on the structure of the DDX5 DEAD domain (PDB ID: 3FE2)^8^ and the amino-acid sequence of DDX41, using the program Molrep^25^. The phases of DEAD_399_ were determined by the program Phaser, using the structure of DEAD_402_ as the search model. The initial models were built using the program PHENIX^26^. For the model building and further refinement, the programs COOT^27^, PHENIX and Refmac5^28^ were used.

### Pull-down assay

The sequence of the sense strand of ISD is as follows: 5′-TACAGATCTACTAGTGATCTATGACTGATCTGTACATGATCTACA-3′. To make the double-stranded bio-ISD, the 5′ biotinylated sense strand of ISD (Eurofin) was mixed with its complementary unlabeled DNA (Eurofin) and annealed. For negative controls and competition assays, unlabeled ATP, ADP, AMPPNP (SIGMA) and cGG (C057-01, BioLog) were used. Bio-ISD, bio-cGG (2′-O- (6-[biotinyl]aminohexylcarbamoyl)-cyclic diguanosine monophosphate, B098-001, BioLog), bio-ATP (N^6^-(6-amino)hexyl-adenosine-5′-triphosphate-biotin, NU-805-BIO, Jena Bioscience) and bio-AMPPNP (2′/3′-O-(2-aminoethyl-carbamoyl)-adenosine-5′-[(β,γ)-imido] triphosphate-biotin, NU-810-BIO, Jena Bioscience), each at a final concentration of 1 μM, were incubated with Dynabeads M-280 Streptavidin (Thermo Fisher Scientific) for 30 min at 4 °C in binding buffer (50 mM Tris-HCl, pH 7.5, 150 mM NaCl, 10% glycerol, 0.5 mM EDTA, 1 mM 2-mercaptoethanol, 0.5% Nonidet P-40). The beads were washed with binding buffer three times, and then were incubated with the proteins for 1 h at 4 °C in binding buffer. The beads were washed with binding buffer three times. The bound proteins were eluted by boiling for 5 min at 95 °C, using SDS-PAGE sample buffer.

### Kinase assay

Purified FL-DDX41 was incubated with the BTK dimer at a 20:1 molar ratio for 16 h at 4 °C, in the presence of 10 mM MgCl_2_ and 1 mM ATP. To isolate the phosphorylated FL-DDX41, the protein was loaded onto a HiTrap Heparin HP column (GE Healthcare), equilibrated with buffer C (used in the purification of DDX41). The protein was eluted with a linear gradient of 100–1,000 mM NaCl. The protein was concentrated with Amicon Ultra Centrifugal Filter Units (MWCO 30 kDa) (Millipore), flash frozen in liquid nitrogen, and stored at −80 °C. The phosphorylation of DDX41 and the self-phosphorylation of BTK were detected by western blotting, using the anti-phosphotyrosine (4G10) antibody (05–321, Upstate Biotechnology), or by SuperSep Phos-tag (Wako). The phosphorylation of FL-DDX41 was detected, although those of DEAD-HELICc and DEAD-HELICc-ZF could not be detected.

### Cell culture, knockdown and reconstitution of DDX41

THP-1 cells (human monocytic leukemia) were maintained in RPMI-1640 medium (Sigma), containing 10% FCS, 100 IU ml^−1^ penicillin G and 100 μg ml^−1^ streptomycin, at 37 °C under a 5% CO_2_ atmosphere. For the stable knockdown of *DDX41*, THP-1 cells were infected with lentiviral particles encoding the shRNA for *DDX41* (Santa Cruz Biotechnology, sc-91765-v), and stable cells were selected by puromycin treatment. To reconstitute the wild-type and mutants of DDX41, the stably knock-downed cells were re-infected by the lentiviral plasmid pCSII-EF-IRES2-Venus, kindly provided by Dr. Hiroyuki Miyoshi (RIKEN BioResource Center), encoding shRNA-resistant DDX41 or its mutants, and selected with blasticidin.

### Cell treatments, qPCR, and immunoblotting

THP-1 cells, in 12-well plates, were stimulated by 1.0 μg ml^−1^ poly(dA:dT) (Invivogen) coated with Lipofectamine 2000 (Invitrogen) for 2 h. For the qPCR, cell lysis and reverse-transcription were performed with a SuperPrep Cell Lysis & RT Kit for qPCR (Toyobo), according to the manufacturer’s instructions. Quantitative real-time PCR was performed with Power SYBR Green PCR Master Mix (Life Technologies), and a Step-One-Plus PCR system (Applied Biosystems) by the ΔΔCT method, using the following oligonucleotides: *IFNB1* sense, 5′-AGGACAGGATGAACTTTGAC-3′, and *IFNB1* antisense, 5′-TGATAGACATTAGCCAGGAG-3′; *IFIT2* sense, 5′-TGGTGGCAGAAGAGGAAGAT-3′, and *IFIT2* antisense, 5′-GTAGGCTGCTCTCCAAGGAA-3′; and *GAPDH* sense, 5′-AGCAACAGGGTGGTGGAC-3′, and *GAPDH* antisense, 5′-GTGTGGTGGGGGACTGAG-3′. For the immunoblotting experiments, the cells were lysed in lysis buffer (50 mM Tris-HCl, pH 7.5, 150 mM NaCl, 1% Triton X-100) containing complete protease inhibitor (Roche), and immunoblotting was performed using the following antibodies: DDX41 (H00051428-M01, Abnova), P-IRF3 (#4947, Cell Signaling Technology), IRF3 (#11904, Cell Signaling Technology), and α-tubulin (Cedarlane).

## Additional Information

**Accession codes**: The coordinates have been deposited in the PDB under the accession codes 5GVR (closed form) and 5GVS (open form).

**How to cite this article**: Omura, H. *et al*. Structural and Functional Analysis of DDX41: a bispecific immune receptor for DNA and cyclic dinucleotide. *Sci. Rep*. **6**, 34756; doi: 10.1038/srep34756 (2016).

## Supplementary Material

Supplementary Information
