# Supplementary material for: Structural and Functional Analysis of DDX41: a bispecific immune receptor for DNA and cyclic dinucleotide
Source: Sci Rep. 2016 Oct 10;6:34756. doi: 10.1038/srep34756 (PMC5056382; doi:10.1038/srep34756)
Supplement: Supplementary Information [file srep34756-s1.pdf]

Supplementary Information – title page

Structural and Functional Analysis of DDX41: a bispecific immune receptor for DNA and cyclic dinucleotide

Hiroki Omura[1], Daisuke Oikawa[2, 3], Takanori Nakane[1], Megumi Kato[1], Ryohei Ishii[1], Ryuichiro Ishitani[1*], Fuminori Tokunaga[2, 3*], and Osamu Nureki[1*]

[1]Department of Biophysics and Biochemistry, Graduate School of Science, The University of Tokyo, 2-11-16 Yayoi, Bunkyo-ku, Tokyo 113-0032, Japan. [2]Laboratory of Molecular Cell Biology, Institute for Molecular and Cellular Regulation, Gunma University, 3-39-15 Showa-machi, Maebashi, Gunma 371-8512, Japan. [3]Department of Pathobiochemistry, Osaka City University, Graduate School of Medicine, 1-4-3 Asahi-machi, Abeno-ku, Osaka 545-8585, Japan.

* Correspondence and requests for materials should be addressed to R. Ishitani (email: ishitani@bs.s.u-tokyo.ac.jp), F. Tokunaga (email: ftokunaga@med.osaka-cu.ac.jp) or O. Nureki (email: nureki@bs.s.u-tokyo.ac.jp)

Table S1. Data Collection and Refinement Statistics

	DDX41 $DEAD_{402}$	DDX41 $DEAD_{399}$
**Data collection**		
Beamline	SLS PXII	SPring-8 BL41XU
Wavelength (Å)	0.979	1.000
Space group	$P3_221$	*I*2
Cell dimensions		
a, *b*, *c* (Å)	81.83, 81.83, 69.57	91.19 50.96 202.60
α, β, γ (°)	90, 90, 120	90, 95.52, 90
Resolution (Å)	40.92–1.50	49.41–2.20
R_{merge}	0.065 (>1.00)	0.202 (0.867)
$I/\sigma I$	18.40 (1.47)	9.3 (2.8)
Completeness (%)	99.9 (99.5)	76.5 (77.5)
Redundancy	10.34 (10.24)	4.4 (4.1)
**Refinement**		
Resolution (Å)	40.92–1.50	49.41–2.20
No. reflections	41,380	34,370
R_{work}/R_{free}	0.181/0.208	0.207/0.275
No. atoms		
Protein	1826	6473
Ligand	9	0
Solvent	114	333
B-factors		
Protein	29.66	24.49
Ligand	35.92	-
Solvent	31.44	21.67
R.m.s. deviations		
Bond lengths (Å)	0.023	0.013
Bond angles (º)	2.18	1.696
Ramachandran plot		
Favored (%)	97.41	96.69
Allowed (%)	2.59	3.19
Outlier (%)	0	0.12

*Highest resolution shell is shown in parentheses.

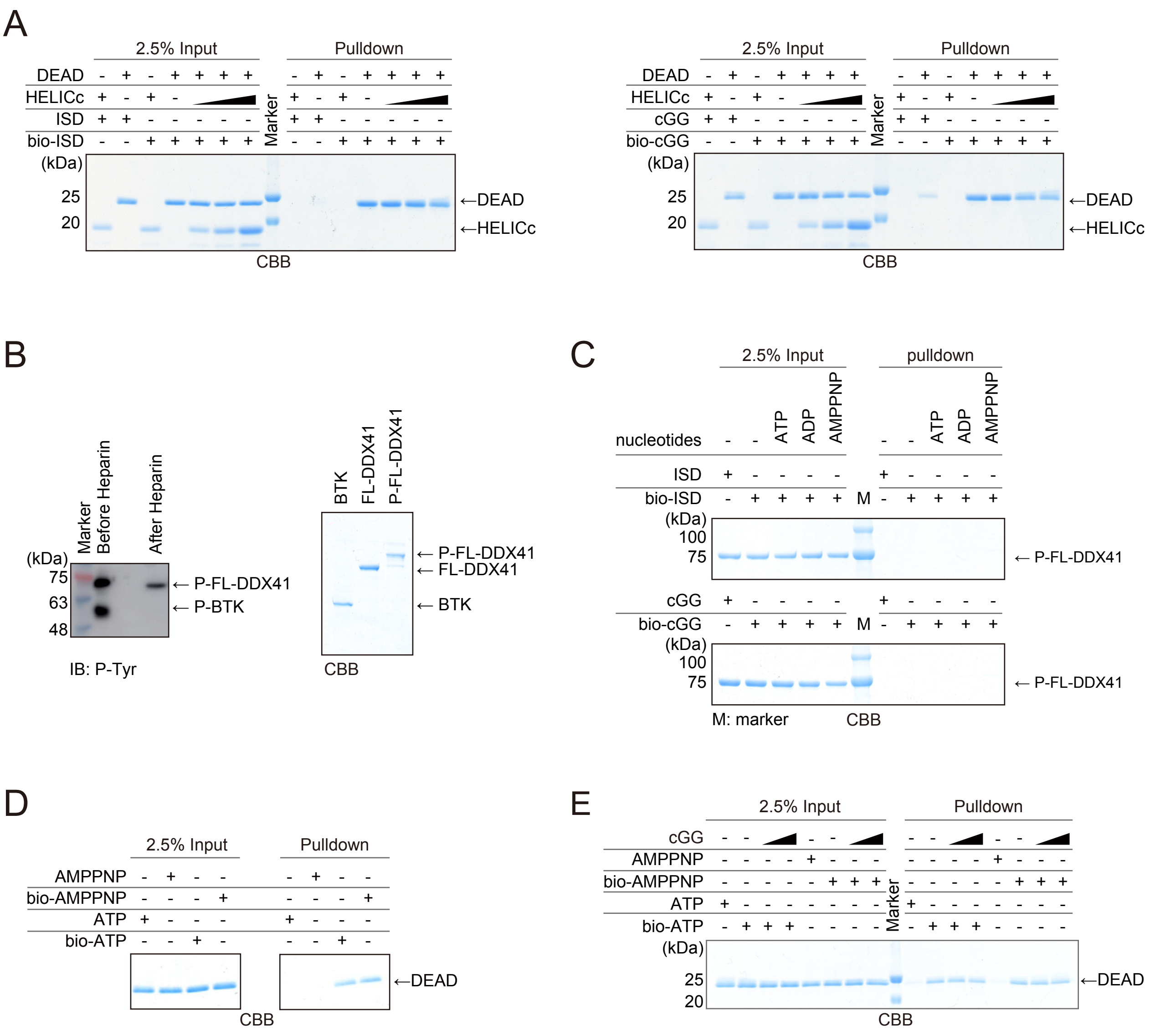

Figure S1. The effects of HELICc, ATP or phosphorylation on dsDNA and CDN binding by DDX41. (A) Pull-down assay of DEAD, using bio-ISD (left panel) and bio-cGG (right panel) in the presence of HELICc. (B) Phosphorylation assay of DDX41 by BTK. FL-DDX41 was mixed with BTK and incubated in the presence of ATP and $MgCl_2$. The phosphorylation of tyrosine was detected by an immunoblot (left panel, “Before Heparin”). Phosphorylated FL-DDX41 (P-FL-DDX41) was isolated using a HiTrap Heparin HP column (left panel, “After Heparin”). The isolated P-FL-DDX41 was detected by a mobility shift assay, using a SuperSep Phos-tag gel (right panel). (C) Pull-down assay of phosphorylated FL-DDX41, using bio-ISD (upper panel) and bio-cGG (left panel). (D) Pull-down assay of DEAD, using bio-ATP or bio-AMPPNP. (E) Pull-down assay of DEAD, using bio-ATP or bio-AMPPNP, in the presence of 0, 1 or 4 μM unlabeled cGG.

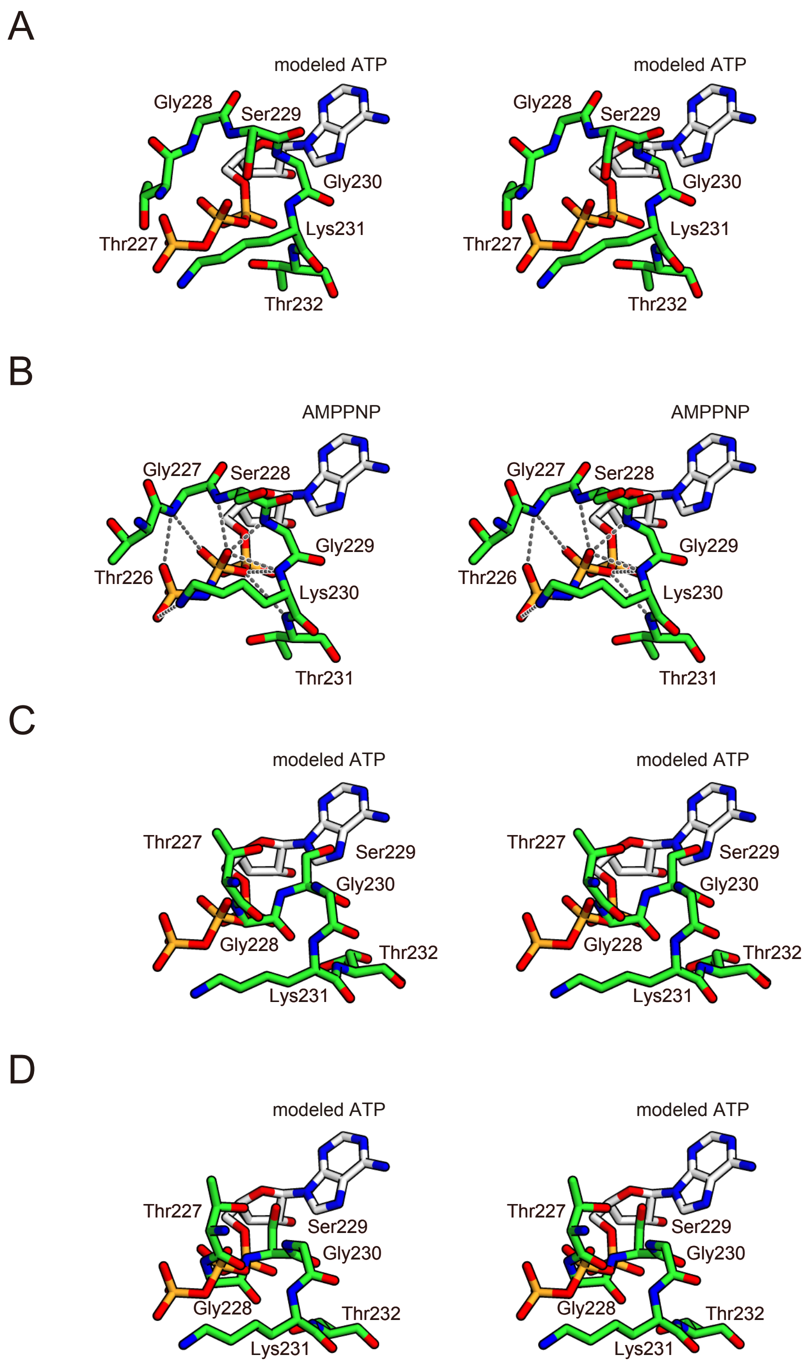

Figure S2. Motif I of the DDX41 closed form interacts with modeled ATP in a similar manner to that of DDX3. (A)(B)(C)(D) The ATP binding mode of motif I. The motif I structures shown (stereo views) are the closed form (A), DDX3 (PDB ID: 5E7M) (B), the open form 1 (C) and the open form 2 (D).

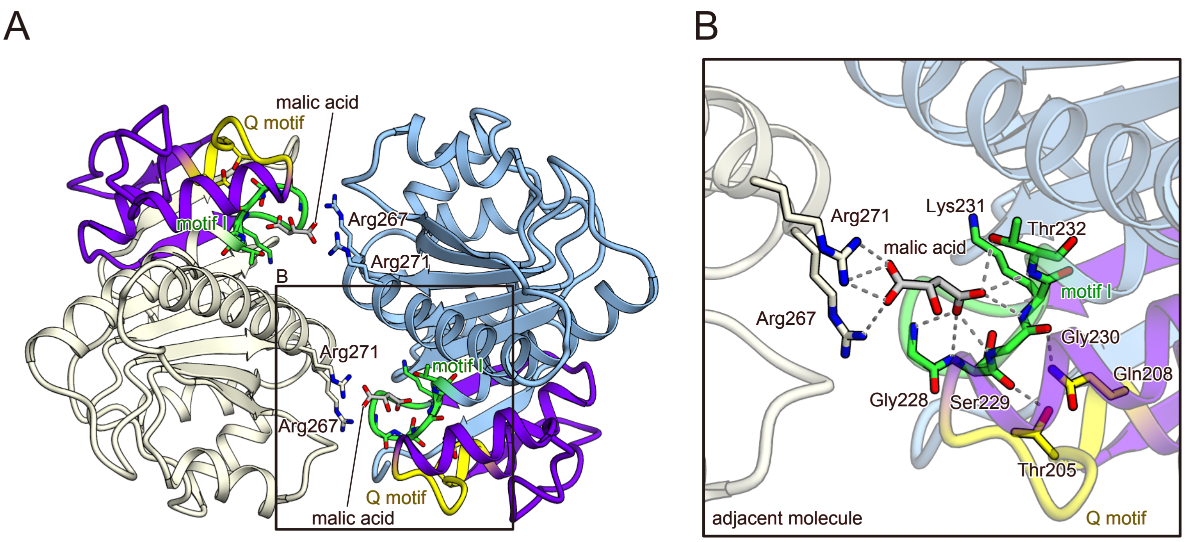

Figure S3. Malic acid binds to motif I of DDX41 by mimicking the phosphate group of ATP. (A) Interaction between the closed form of DDX41 and malic acid. The adjacent molecule of DDX41 is colored beige. (B) The details of the interaction between DDX41 and malic acid.

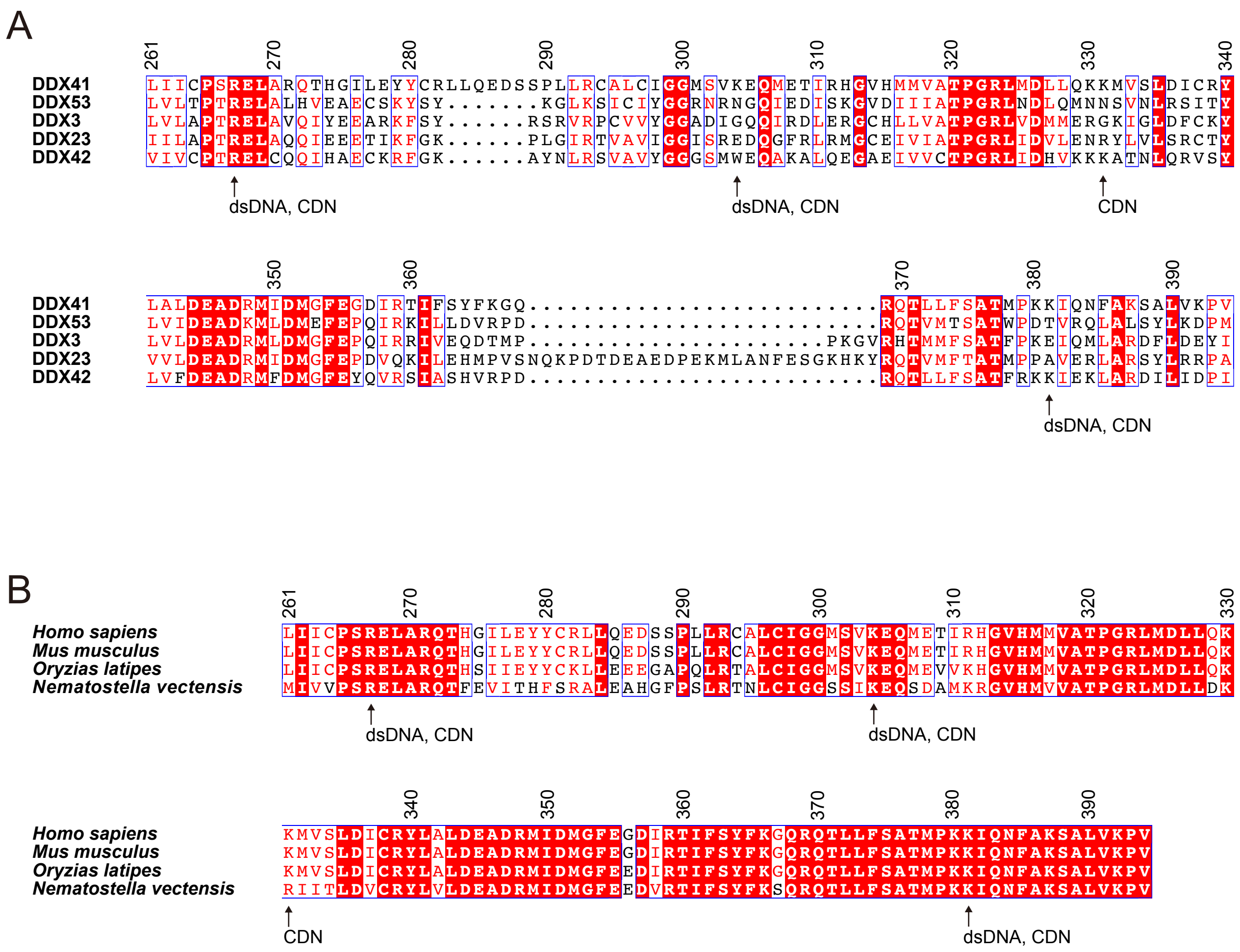

Figure S4. Sequence alignment of DDX41 with its orthologues and paralogues. (A) Amino-acid sequence of the residues near the putative dsDNA/CDN binding sites of DDX41 and its paralogues. Residues critical for dsDNA or CDN binding are indicated. Sequences shown are human DDX41 (gene accession number: 21071032), DDX3 (301171467), DDX23 (41327771), and DDX42 (15930131). (B) Phylogenetic alignment of DDX41. The sequences are DDX41 derived from *Homo sapiens* (gene accession number: 21071032), *Mus musculus* (270047502), *Oryzias latipes* (Japanese rice fish, 432879043) and *Nematostella vectensis* (starlet sea anemone, 156406955).

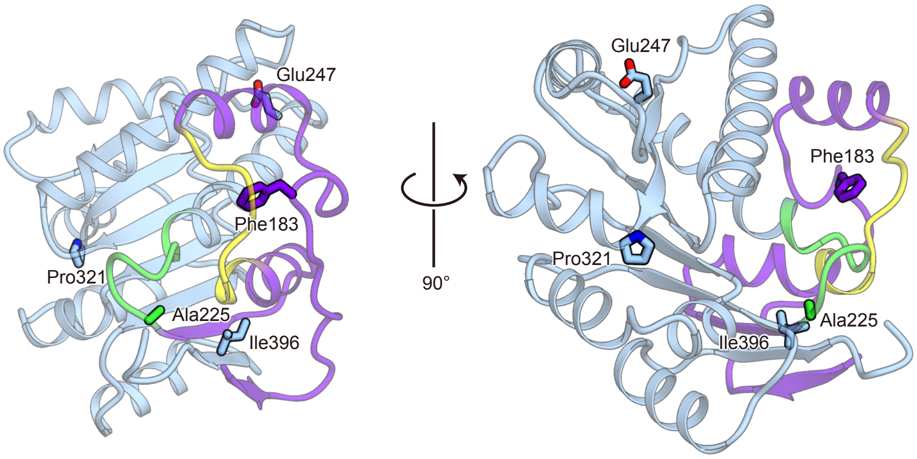

Figure S5. Residues mutated in AML patients form the inside core of the DEAD domain. The residues mutated in AML patients are mapped on the structure of the DEAD domain.

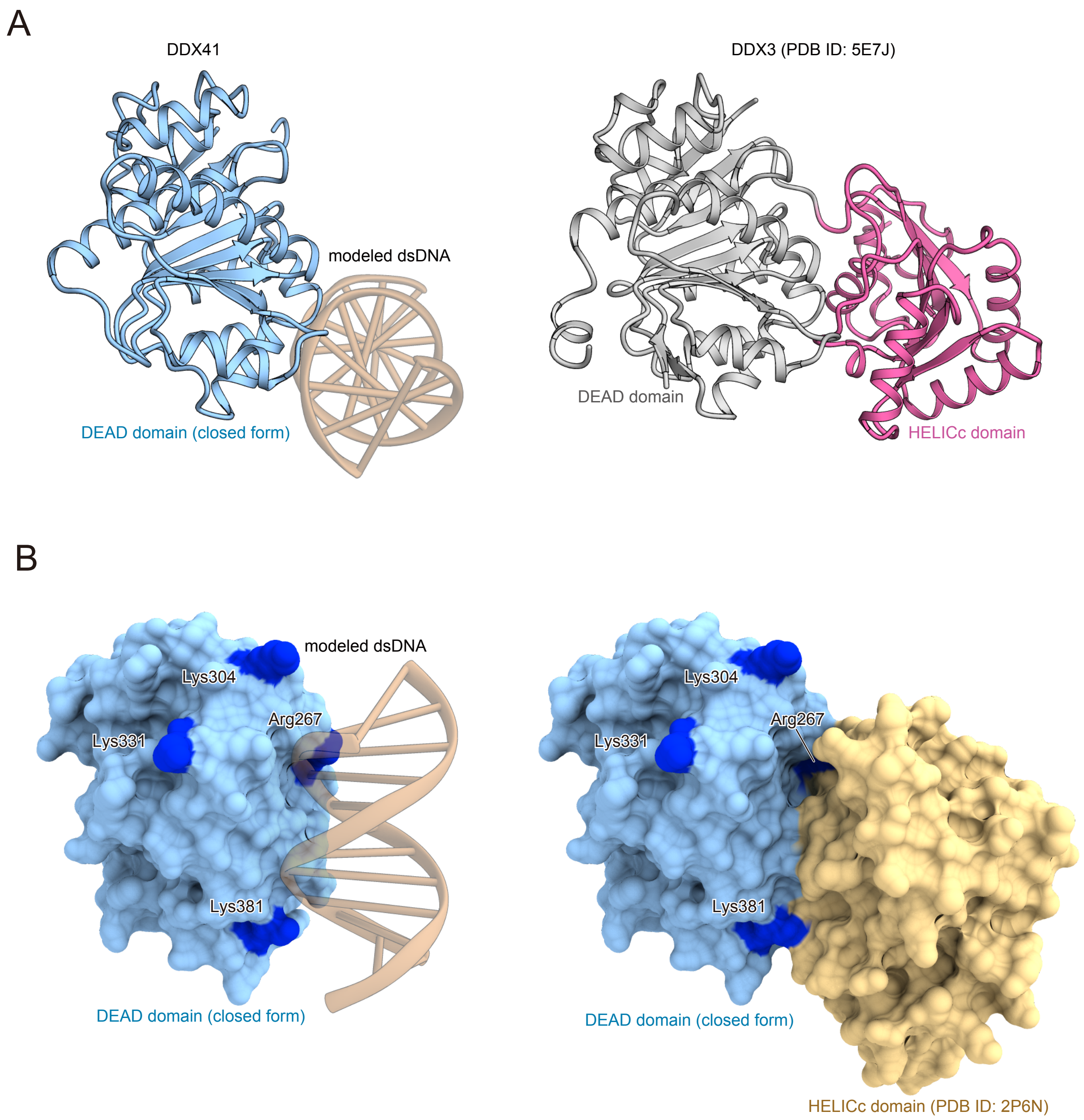

Figure S6. Models of HELICc domain-mediated inhibition of dsDNA and CDN binding by the DEAD domain. (A) Docking model of DDX41 bound to dsDNA (left panel), and the structure of DDX3 DEAD-HELICc (PDB ID: 5E7J) (right panel). The DEAD domain of DDX41 is colored sky blue. The DEAD domain and the HELICc domain of DDX3 are colored silver and magenta, respectively. The two structures are in the same orientation. (B) Docking model of DDX41 bound to dsDNA (left panel) and model of DDX41 DEAD-HELICc (right panel), in the molecular surface representation. The latter model is based on the structure of DDX3 (PDB ID: 5E7J). Residues in the putative dsDNA/CDN-binding surface are colored blue. The DEAD domain and the HELICc domain (PDB ID: 2P6N) are colored sky blue and orange, respectively. The two models are in the same orientation.

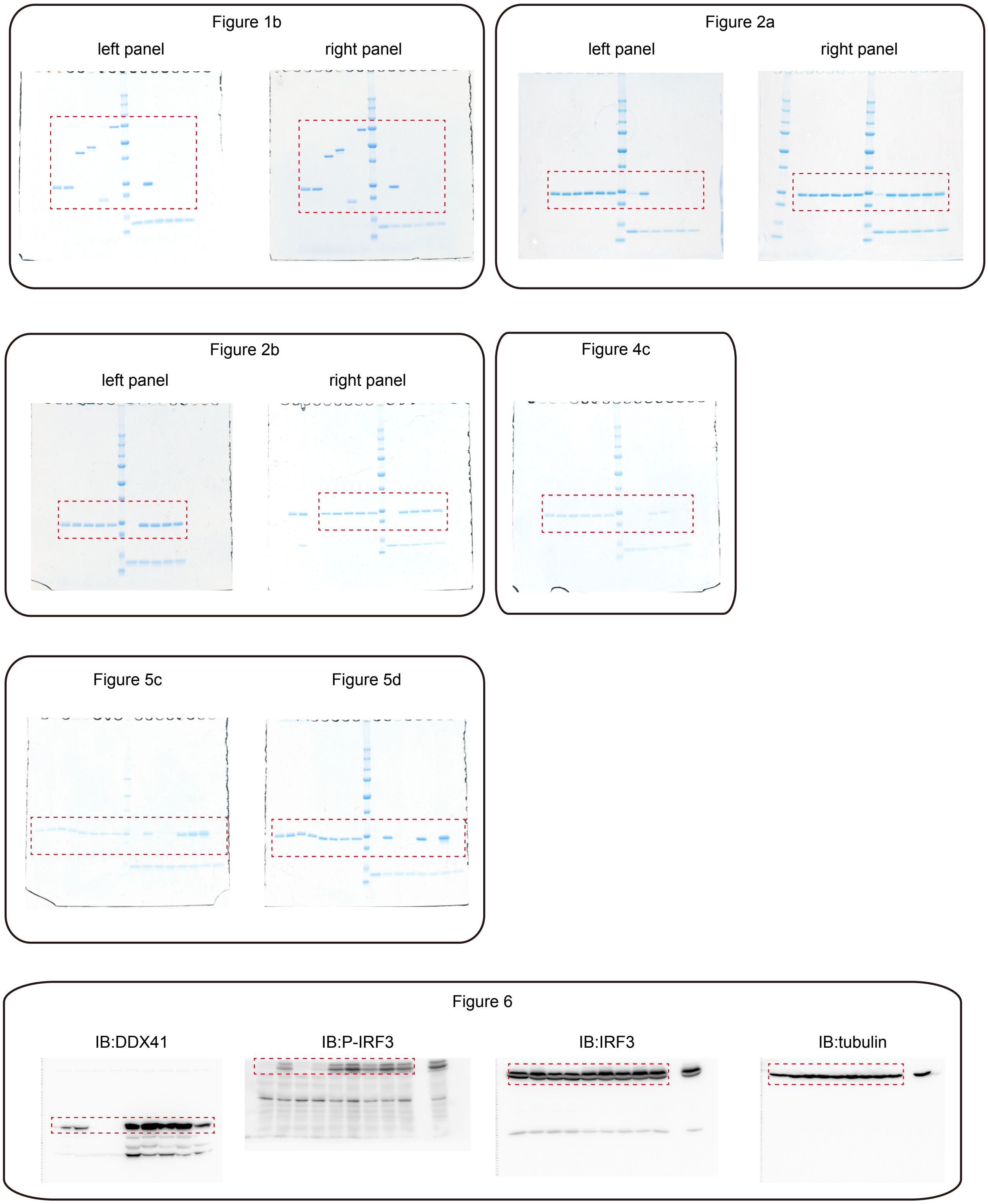

Figure S7. Full-length gels and blots. Uncropped gels and blots are shown. The red boxes indicate the cropped regions.
